# A wandering spleen with 720° torsion and persistent ascending and descending mesocolon in a 5-year-old Syrian male: A case report^☆^

**DOI:** 10.1016/j.ijscr.2023.108319

**Published:** 2023-05-13

**Authors:** Hiyam Ahmad, Hussein Hamdar, Ali Alakbar Nahle, Nafiza Martini, Zaed Alkhatib

**Affiliations:** a Damascus University, Faculty of Medicine, Damascus, Syrian Arab Republic; b Stemosis for Scientific Research, Damascus, Syrian Arab Republic; c Ibn Al-Nafees Hospital, Damascus, Syrian Arab Republic

**Keywords:** Wandering spleen, Spleen torsion, Persistent mesocolon, Case report

## Abstract

**Introduction and importance:**

The spleen is normally found in the left hypochondrium and it is fixed in its place by numerous suspensory ligaments. When the ligaments are elongated or abnormally developed, it causes a rare medical condition called Wandering spleen. A persistent ascending and descending mesocolon is also a congenital anomaly, resulting from the failure of fusion of the primitive dorsal mesocolon.

**Case presentation:**

Herein, a 5-year-old male child with sudden and acute onset of abdominal pain, constipation, nausea, tachycardia, and low urine output, imaging and blood tests revealed evidence of intestinal obstruction and normocytic anemia and neutrophilia. A laparotomy revealed persistent ascending and descending mesocolon, with a torsioned vascular pedicle of the spleen, resulting in splenomegaly and infarction. The surgeon successfully derotated the torsioned pedicle and performed a splenectomy. The patient had an uneventful postoperative course and was discharged without complications.

**Clinical discussion:**

This case could be asymptomatic and the diagnosis is incidental or it could be presented with ambiguous symptoms. The differential diagnosis of WS varies according to the clinical presentation and the associated complication. For instance, in the case of WS torsion and acute presentation, the differential diagnosis is ovarian torsion, acute appendicitis, and intestinal obstruction.

Currently, surgery is the only suggested treatment option even in asymptomatic patients as well.

**Conclusion:**

This case of a Wandering Spleen is associated with a persistent ascending and descending mesocolon, suggesting that there may be certain risk factors. Therefore, we suggest making more research about wandering spleen in association with persistent mesocolon.

## Highlights

• Wandering spleen is usually present in children and young adults especially women of reproductive age with an incidence rate of less than 0.2%.
• This condition could be asymptomatic and the diagnosis is incidental or it could be presented with ambiguous symptoms. Therefore, it is important to emphasize the need to pay attention to these cases.
• Currently, surgery is the only suggested treatment option for the wandering spleen and it is required even in asymptomatic patients as well.
• Spleen preservation by splenopexy is the recommended surgery to avoid post-splenectomy sepsis syndrome in the future. Also, it is recommended to take vaccinations after the splenectomy surgery.
• We suggest to make more research about wandering spleen in association with persistent mesocolon.

## 1 Introduction

The spleen is typically situated in the left hypochondrium and anchored in place by various suspensory ligaments. However, elongated or abnormal development of these ligaments can cause the spleen to become more mobile, leading to a rare medical condition known as Wandering spleen [1]. Wandering spleen is most commonly found in children and young adults, particularly women of reproductive age, with a low incidence rate of less than 0.2 % [2,3]. This condition may have no symptoms and may be discovered incidentally or may present with vague symptoms, a palpable abdominal mass or acute abdomen [1,4,5]. Diagnosis can be challenging, particularly in children, due to the nonspecific presentation. However, the diagnosis of Wandering spleen is usually straightforward, and helpful investigations include plain X-ray, Doppler ultrasonography, computerized tomography (CT), magnetic resonance imaging (MRI), scintigraphy, and splenic angiogram [[6], [7], [8]]. The optimal treatment for ectopic spleen is open or laparoscopic surgery, with splenopexy being the preferred option in the absence of infarction, splenomegaly, or hypersplenism, and splenectomy being required when these complications are present [4]. A persistent mesocolon is an embryological abnormality that occurs when the mesentery of the ascending and descending colons fails to fuse with the posterior lateral parietal peritoneum after five months of gestation [9]. In this case, a five-year-old boy was found to have both Wandering spleen and a persistent mesocolon during surgery.

## 2 Case presentation

A 5-year-old male child presented with sudden, diffuse abdominal pain predominantly in the hypogastric region that had persisted for 12 h. He also reported experiencing constipation and nausea, but not vomiting. The patient had previously experienced episodes of brief-duration abdominal pain. He appeared drowsy and lethargic upon examination.

During the physical examination, vital signs revealed tachycardia (pulse rate of 164 beats per minute), a respiratory rate of 26 breaths per minute, a temperature of 38C°, and low urine output (9 ml per hour). His abdomen was distended and moved with respiration. Generalized tenderness was present, especially in the suprapubic area, where a palpable, firm mass was also noted. There was resonance upon percussion, and bowel sounds were increased upon auscultation. A digital rectal examination was unremarkable. Blood workup revealed normocytic anemia and neutrophilia.

**Fig. 1:**
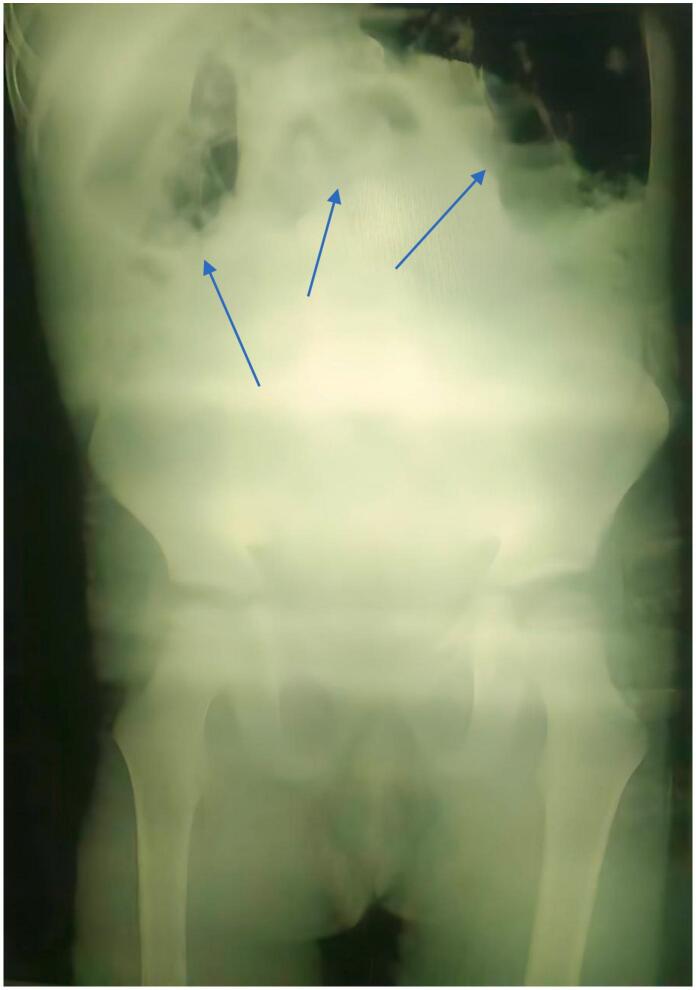
Abdominal X-ray showing intestinal obstruction.

Abdominal X-ray imaging revealed evidence of intestinal obstruction (Fig. 1). Ultrasound imaging was not available, and an abdominal CT scan was not performed due to the patient's shock condition.

**Fig. 2:**
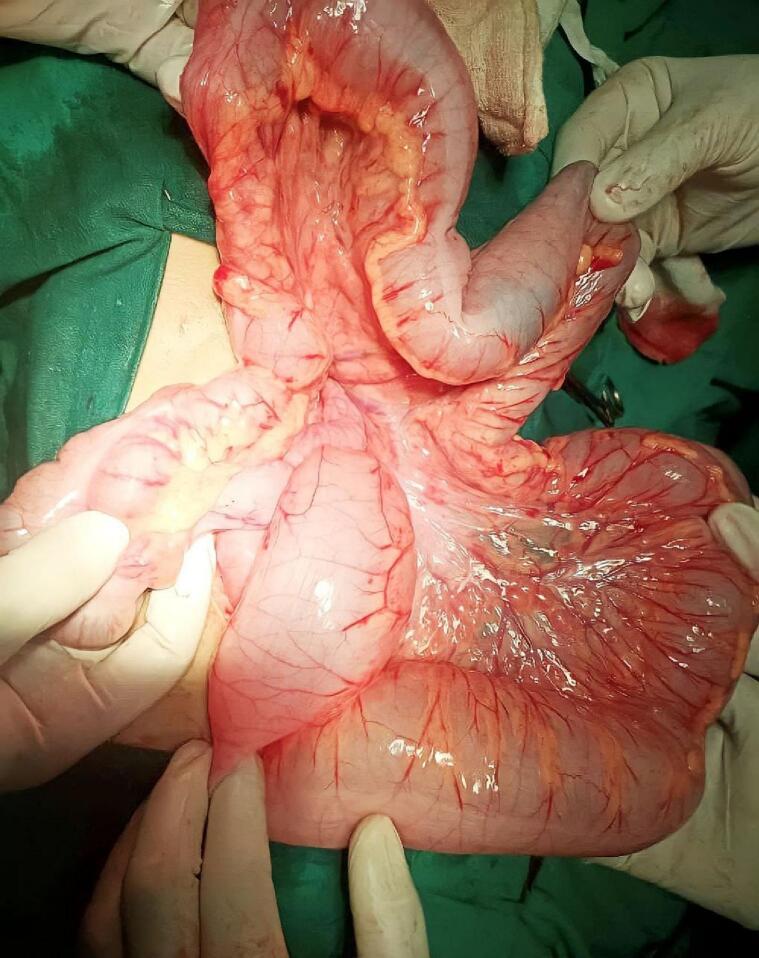
Dilation in the loops of intestine was seen during surgery with an absence mesocolon fixation of ascending and descending colon to the posterior abdominal wall with the peritoneum.

The patient was administered isotonic crystalloid solutions, and a nasogastric tube and urinary catheter were inserted. Antibiotics (cefazolin) were also administered to the patient. After stabilization, a laparotomy was performed, revealing dilation in the small and large intestine loops reaching the descending colon, with an absence of mesocolon fixation of the ascending and descending colon to the posterior abdominal wall with the peritoneum (Fig. 2). This left us with a concomitant diagnosis of persistent ascending and descending mesocolon.

**Fig. 3:**
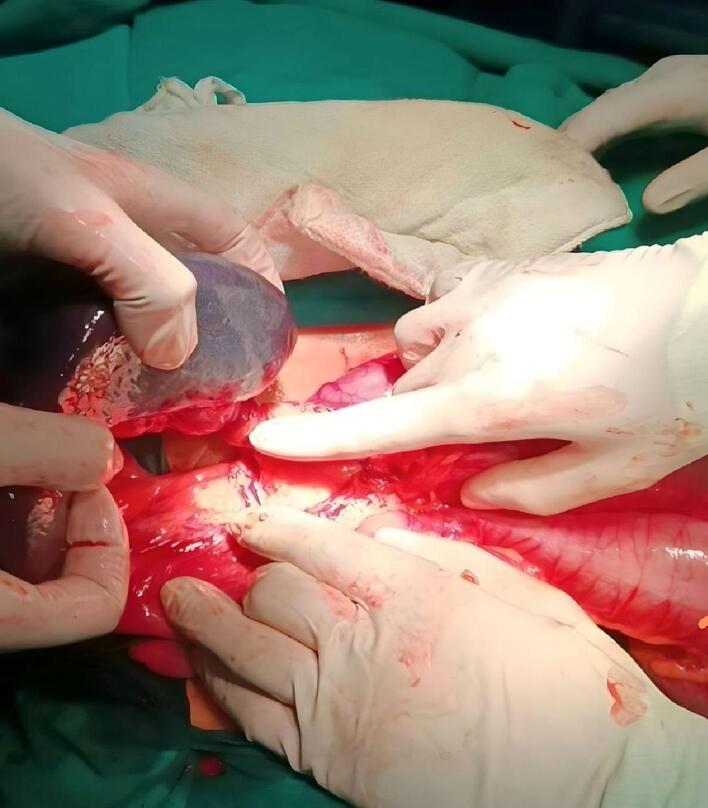
Torsioned vascular pedicle during surgery.

**Fig. 4:**
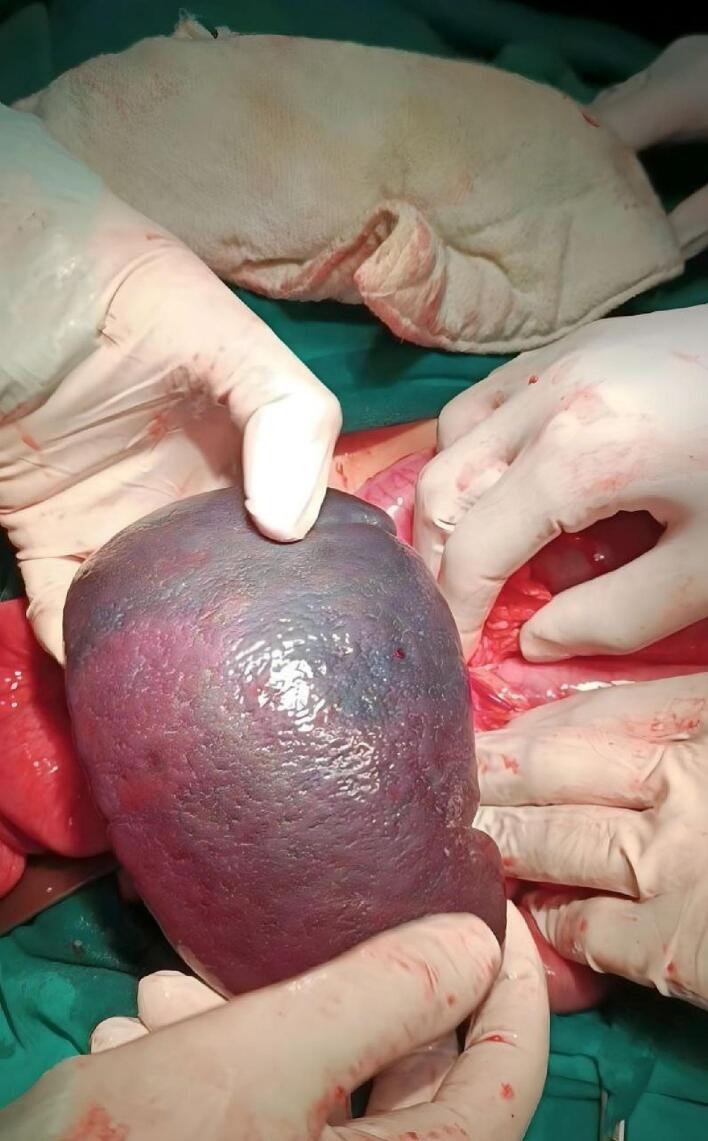
Segmental infraction and infraction spots seen on the spleen during surgery.

The spleen was identified in the pelvic cavity, pressing on the colon. The vascular pedicle was found to be torsioned 720° counterclockwise, with spots of infraction on the spleen (Fig. 3, Fig. 4). The surgeon successfully derotated the torsioned pedicle. Due to the significant spots of infraction on the spleen and splenomegaly, the surgeon decided to perform a splenectomy (Video 1).

The patient's postoperative course was uneventful, and he was discharged after 48 h without any complications. It was recommended that he receive vaccinations 3 weeks after the splenectomy surgery.

## 3 Discussion

Wandering spleen (WS) with torsion is a rare condition that accounts for less than 0.2 % of all splenectomies [10]. It has a bimodal age incidence, affecting children under 10 years old and women of reproductive age, with an average of 25.2 years [11,12]. However, within the first 10 years of life, it may have the same incidence rate for both sexes [13]. The etiology of WS is multifactorial and can be categorized into acquired and congenital causes [14]. The suspensory ligaments of the spleen are classified into various types, including gastrosplenic, splenorenal, splenocolic, splenophrenic, and pancreatico-splenic ligaments. The absence or excessive laxity of these ligaments can result in a hypermobile spleen [14]. Acquired causes of WS include conditions that can damage or weaken the suspensory ligaments, such as trauma and connective tissue disorders [14]. In contrast, congenital etiology is considered the most common cause of WS, especially in pediatric patients [10], and is characterized by the absence or maldevelopment of one or more ligaments, resulting in a hypermobile spleen. This is due to incomplete fusion of the mesogastrium and the posterior abdominal wall during the second month of pregnancy [[15], [16], [17]]. In our case, despite the presence of twisted vascular pedicle, no suspensory ligaments were identified along with the WS, and there was no history of trauma or any associated form of acquired causes, leading to a suspicion of congenital WS. This suspicion was further supported by the presence of a persistent ascending and descending mesocolon in the patient.

A persistent ascending and descending mesocolon is a congenital anomaly that arises from the failure of fusion between the primitive dorsal mesocolon and the parietal peritoneum during the fifth month of gestation [18]. In this case, we present the first reported occurrence of a WS in association with a persistent ascending and descending mesocolon, indicating potential underlying risk factors for this association. However, due to limited financial resources, further investigation of this condition, such as complete genetic investigation, was not feasible.

Wandering spleen (WS) can manifest in various clinical presentations. It may remain asymptomatic throughout an individual's lifetime [19]. Although the majority of adults with WS are asymptomatic and diagnosed incidentally through physical examination or imaging studies for other reasons [14,20], pediatric patients typically exhibit acute abdominal pain when complicated by intestinal obstruction or splenic torsion. The degree of torsion can determine the severity of pain, and severe torsion can lead to splenic infarction, resulting in an acute abdomen [10]. In our case, the child experienced severe abdominal pain due to extreme torsion, leading to splenic infarction. Studies have demonstrated that elongated twisting of more than 180° can cause splenic infarction [21,22], and in this case, the spleen was twisted to a degree of 720°. WS can result in other complications, such as gastric volvulus, variceal hemorrhage, and acute pancreatitis [23,24]. The differential diagnosis of WS varies depending on the clinical presentation and associated complications. For example, in cases of WS torsion and acute presentation, differential diagnoses may include ovarian torsion, acute appendicitis, and intestinal obstruction [25].

The diagnosis of WS can be confirmed by various diagnostic techniques, such as Doppler ultrasound and magnetic resonance imaging [26]. Ultrasound imaging with duplex scanning is an early imaging technique that can show the location of the wandering spleen with concurrent bowel replacement in the left upper quadrant [27]. However, in our department, this technique was not available. CT scan remains the preferred procedure, as it may demonstrate the efficacy of the organ's blood flow and the viability of splenic parenchyma [28]. The absence of the spleen from its correct location and the presence of an aberrant mass in the abdomen or pelvis are the most distinguishing features [29]. According to a previous study, an ultrasound of the abdomen on a patient with WS, showed a uniform mass resembling the spleen in the midline of the abdomen. Nevertheless, Doppler imaging yielded negative results in detecting any blood flow, and no splenic tissue was observed in the left upper quadrant. Further radiological evaluation using CT scan revealed additional features of WS, such as splenic infarction, twisting of the spleen, swirling of splenic vessels, and an abnormal spleen position [25]. However, in our case, due to the patient's critical condition and shock state, a CT scan was not performed. Instead, an urgent abdominal X-ray was conducted, revealing intestinal obstruction followed by immediate surgical intervention.

Currently, surgery is the only recommended treatment option for WS [30]. Even in the absence of symptoms, surgical intervention is necessary in WS due to the high rate of associated complications [25,31]. There exist two surgical procedures - splenectomy and splenopexy - to address a wandering spleen. Splenectomy involves the complete removal of the spleen, while splenopexy seeks to preserve the spleen [32]. Splenectomy is recommended when the spleen is enlarged, infarcted, ruptured, or exhibiting signs of hypersplenism and can be performed laparoscopically or via laparotomy [[33], [34], [35]]. However, in all other situations, spleen preservation via splenopexy is the preferred surgical approach to avoid post-splenectomy sepsis syndrome in the future [36]. In our case, a splenectomy was performed due to the presence of both infarction spots and segmental infarction of the spleen during surgery, and the procedure was done via laparotomy due to the lack of instruments in our department. After undergoing surgery, children must receive post-splenectomy vaccines against pathogens such as influenza and meningococcus due to affected immunity [25,32]. This is consistent with our case, where the patient received vaccination three weeks after splenectomy.

This case report has been reported in line with the SCARE criteria [37].

## 4 Conclusion

Wandering spleen is a very rare case. In many cases it can exist in asymptomatic patients or with emergence symptoms. Therefore, it is important to emphasize the need to pay attention to these cases. In our case, a splenectomy was performed due to infarction spots seen on the spleen. In addition, splenectomy was done via laparotomy instead of laparoscopy due to the lack of instruments in our department. However, in all other situations, spleen preservation by splenopexy is the recommended surgical approach to avoid post-splenectomy sepsis syndrome in the future. Also, it is recommended to take vaccinations after the splenectomy surgery.

***Finally***, this case of a Wandering Spleen is associated with a persistent ascending and descending mesocolon, suggesting that there may be certain risk factors contributed to this association. Therefore, we suggest making more research about wandering spleen in association with persistent mesocolon.

The following is the supplementary data related to this article.

Supplementary Video 1 The surgeon derotated the torsioned pedicle and decided to perform a splenectomy. Supplementary Video 1

## Abbreviations

CT: computerized tomography
MRI: magnetic resonance imaging
WS: Wandering spleen

## Ethics approval and consent to participate

Ethical approval was also taken from the Faculty of Medicine at Damascus University.

## Funding

None.

## CRediT authorship contribution statement

**HA & HH & AAN** are co-first authors. All contributed equally in this paper.

**HA** contributed to drafting, editing, reviewing, & bibliography. The author reviewed and accepted the paper.

**HH** contributed to drafting, editing, reviewing, & bibliography. The author reviewed and accepted the paper.

**AAN** contributed to drafting, editing, reviewing, & bibliography. The author reviewed and accepted the paper.

**NM** is the corresponding author. He also contributed to drafting, editing, reviewing, & bibliography. The author reviewed and accepted the paper.

**ZA** is the supervisor general surgeon who operated the patient and reported the data of the case, contributed to editing and reviewing the final version. The author reviewed and accepted the paper.

All authors reviewed and accepted the paper.

## Guarantor

Dr. Zaed Alkhatib.

## Research registration number

This study is a case report, so we can't make the registration it as a trial.

## Consent for publication

Written informed consent was obtained from the patient's parents for publication of this case report and any accompanying images and videos. A copy of the written consent is available for review by the editor of this journal on request.

## Provenance and peer review

Not commissioned, externally peer-reviewed.

## Conflict of interest statement

No conflict of interest.

## References

[1] Faridi M.S., Kumar A., Inam L., Shahid R. (Nov. 1, 2014). Wandering spleen—a diagnostic challenge: case report and review of literature. Malays. J. Med. Sci..

[2] Masroor M., Sarwari M.A. (2021 Dec 1). Torsion of the wandering spleen as an abdominal emergency: a case report. BMC Surg..

[3] Alghamdi R., Alzahrnai A., Alosaimi A., Albabtain I. (2021 Jun 1). Infarcted wandering spleen: a case report from Saudi Arabia. J. Surg. Case Rep..

[4] Blouhos K., Boulas K.A., Salpigktidis I., Barettas N., Hatzigeorgiadis A. (2014). Ectopic spleen: an easily identifiable but commonly undiagnosed entity until manifestation of complications. Int. J. Surg. Case Rep..

[5] (2019 Jan 1). Emergency laparoscopic splenectomy for torsion of wandering spleen in a geriatric patient: a case report. Int. J. Surg. Case Rep..

[6] Ben Ely A., Zissin R., Copel L., Vasserman M., Hertz M., Gottlieb P. (2006 Nov 1). The wandering spleen: CT findings and possible pitfalls in diagnosis. Clin. Radiol..

[7] Magowska A. (2013 Mar). Wandering spleen: a medical enigma, its natural history and rationalization. World J. Surg..

[8] Flores-Ríos E., Méndez-Díaz C., Rodríguez-García E., Pérez-Ramos T. (2015 Oct 1). Wandering spleen, gastric and pancreatic volvulus and right-sided descending and sigmoid colon. J. Radiol Case Rep..

[9] Balthazar E.J. (1977). Congenital positional anomalies of the colon: radiographic diagnosis and clinical implications - II. Abnormalities of fixation. Gastrointest. Radiol..

[10] Taylor C.S., Howard-Claudio C. (2019). Wandering spleen with splenic torsion in a child with DiGeorge syndrome. Radiol. Case Rep..

[11] Flores-Rios E., Mendez-Diaz C., Rodriguez-Garcia E. (2015). Wandering spleen, gastric and pancreatic volvulus and right-sided descending and sigmoid colon. J. Radiol. Case Rep..

[12] Seif Amir Hosseini A., Streit U., Uhlig J. (2019). Splenic torsion with involvement of pancreas and descending colon in a 9-year-old boy. BJR Case Rep..

[13] Di Crosta I., Inserra A., Gil C.P. (2009). Abdominal pain and wandering spleen in young children: the importance of an early diagnosis. J. Pediatr. Surg..

[14] Nastiti N.A., Niam M.S., Khoo P.J. (2019). Emergency laparoscopic splenectomy for torsion of wandering spleen in a geriatric patient: a case report. Int. J. Surg. Case Rep..

[15] Allen K.B., Gay BBJr, Skandalakis JE. (1992). Wandering spleen: anatomic and radiologic considerations. South. Med. J..

[16] Gligorievski A. (2017). Ectopic spleen presenting as pelvic mass. Trends Med..

[17] Lourdusamy V., Patel D., Docobo R. (2018). The importance of recognizing wandering spleen as a cause of recurrent acute pancreatitis. Case Rep. Gastrointest. Med..

[18] Tsuruta A., Kawai A., Oka Y., Okumura H., Matsumoto H., Hirai T., Nakamura M. (2014). Laparoscopic right hemicolectomy for ascending colon cancer with persistent mesocolon. World J. Gastroenterol..

[19] Hamdy O., Yousry M., Saleh G.A. (2021). An infarcted wandering spleen leading to a sigmoid volvulus presenting with acute large bowel obstruction: a case report. Ann. R. Coll. Surg. Engl..

[20] Khan D.B., Khandwala K., Abbasi S.U. (2018). Torsion of wandering spleen with infarction. Cureus.

[21] Faridi M.S., Kumar A., Lubna I.N., Shahid R. (2014). Wandering spleen-a diagnostic challenge: case report and review of literature. Malays. J. Med. Sci..

[22] Buehner M., Baker M.S. (1992). The wandering spleen. Surg Gynecol Obstet.

[23] Qazi S.A., Mirya S.M., Muhammad A.A. (2004). Wandering spleen. Saudi J. Gastroenterol..

[24] Rosen A., Nathan H., Luciansky E., Sayfan J. (1988). The lienorenal ligament and the tail of the pancreas: a surgical anatomical study. Pancreas.

[25] Sharma A., Salerno G. (2014). A torted wandering spleen: a case report. J. Med. Case Rep..

[26] Lahiri Somdatta, Dasgupta Nabanita, Mondal Aftab-ud-din (2010). A case of splenic torsion and rupture presenting as ruptured ectopic pregnancy. J. Surg. Case Rep..

[27] Karmazyn B., Steinberg R., Gayer G., Grozovski S., Freud E., Kornreich L. (2005). Wandering spleen-the challenge of ultrasound diagnosis: report of 7 cases. J. Clin. Ultrasound.

[28] Gayer G., Zissin R., apter S, Atar E, Portnoy O, Itzchak Y. (2001). CT findings in congenital anomalies of the spleen. Br. J. Radiol..

[29] Ben Elya A., Zissinb R., Copela L., Vassermana M., Hertzc M., Gottlieba P., Gayera G. (2006). The wandering spleen: CT findings and possible pitfalls in diagnosis. Clin. Radiol..

[30] Dawson J.H., Roberts N.G. (1994 Jun). Management of the wandering spleen. Aust. N. Z. J. Surg..

[31] Lane T.M., South L.M. (1999). Management of a wandering spleen. J. R. Soc. Med..

[32] Stringel G., Soucy P., Mercer S. (1982). Torsion of the wandering spleen: splenectomy or splenopexy. J. Pediatr. Surg..

[33] Calik A., Bilgin Y., Kucuktulu U., Cinel A. (1996). Intestinal obstruction caused by splenic volvulus: report of a case. Surg. Today.

[34] Blouhos K., Boulas K.A., Salpigktidis I., Barettas N. (2014). Case report—open access International Journal of Surgery Case Reports ectopic spleen: an easily identifiable but commonly misdiagnosed entity until manifestation of complications in adulthood. Int. J. Surg. Case Rep..

[35] Ayuning N., Niam M.S., Jhiew P. (2019). Case report—open access International Journal of Surgery Case Reports emergency laparoscopic splenectomy for torsion of wandering spleen in a geriatric patient: a case report case report—open access. Int. J. Surg. Case Rep..

[36] M. Jawad, M.H. Yusuf, K.A. Al Doaibel, F.M. Nesaif, A.S. Alharbi, Wandering spleen: a rare case from the emergency department, Cureus. 15 (n.d.) e33246. 10.7759/cureus.33246. PMC9890613 36741617

[37] Agha R.A., Franchi T., Sohrab C., Mathew G., Kirwan A., Thomas A. (2020). The SCARE 2020 guideline: updating consensus Surgical Case Report (SCARE) guidelines. Int. J. Surg..

